# Reproductive trade-offs of the estuarine copepod *Eurytemora affinis* under different thermal and haline regimes

**DOI:** 10.1038/s41598-021-99703-0

**Published:** 2021-10-11

**Authors:** Anissa Souissi, Jiang-Shiou Hwang, Sami Souissi

**Affiliations:** 1 Université de Lille, CNRS, Université du Littoral Côte d’Opale, IRD, UMR 8187 LOG, Laboratoire d’Océanologie Et de Géosciences, Station Marine de Wimereux, 59000 Lille, France; 2 Univ. Littoral Côte d’Opale, UMR 1158 BioEcoAgro, TERRA Viollette, USC Anses, INRAe, Univ. Lille, Univ. Artois, Univ. Picardie Jules Verne, Univ. Liège, 62200 Yncréa, Boulogne-sur-Mer France; 3 Institute of Marine Biology, National Taiwan Ocean University, 20224 Keelung, Taiwan; 4 Center of Excellence for Ocean Engineering, National Taiwan Ocean University, Keelung, 20224 Taiwan; 5 Center of Excellence for the Oceans, National Taiwan Ocean University, Keelung, 20224 Taiwan

**Keywords:** Ecology, Zoology

## Abstract

Copepod females invest a quantity of resources in their reproduction. Depending on several biotic and abiotic factors and their evolutionary history a trade-off can be commonly observed between producing a large number of smaller offspring or a small number of larger offspring. In this study, a multi-generational approach was applied to determine whether a trade-off between clutch size and egg size existed in the copepod *Eurytemora affinis* under different controlled conditions of temperature and salinity*.* This protocol was based on the follow-up of reproductive (Clutch Size ‘CS’, Egg Diameter ‘ED’) and morphological (Prosome Length ‘PL’) traits during several generations. Copepods were acclimated to cold (7 °C) and warm (20 °C) temperatures, and then their reproductive output was tested at the higher temperature of 24 °C. CS and ED were positively correlated to PL, so as a first step linear regressions between each reproductive trait and female PL were performed. The residuals from the regression lines of CS and ED with PL were calculated to remove the effect of female size. When the normalized data (residuals) of CS and ED plotted together a negative relationship between egg size and egg number revealed the existence of a trade-off. Copepod populations initially acclimated to cold temperature are commonly characterized by relatively smaller CS and larger ED. Conversely, warm temperature adapted females produced relatively larger CS and smaller ED. After transfer to a temperature of 24 °C, the ED did not change but the CS showed high variability indicating stressful conditions and no trade-off was observed. These observations suggest that *E. affinis* is able to modulate its reproduction depending on the encountered temperature. It seems that this copepod species can shift between a K- and an r-strategy in response to colder or warmer conditions. In a late winter-early spring like cold temperature, copepod females seem to invest more on offspring quality by producing relatively larger eggs. This ecological strategy ensures a high recruitment of the spring generation that is responsible for the strength of the maximum population size usually observed in late spring-early summer (May–June). To the contrary, at summer-like temperature, where the population density decreases significantly in the Seine estuary, copepod females seem to switch from K to r strategy by favoring offspring number compared to offspring size. Finally, the use of a higher temperature of 24 °C seems to disrupt the observed reproductive trade-off even after several generations. These results suggest that a switching between K- or r-strategy of *E. affinis* depends highly on temperature effects. The effect of salinity increase during a summer-like temperature of 20 °C as well as after transfer to 24 °C decreased PL and CS but the ED did not change significantly.

## Introduction

Reproductive strategies are among the most important outcomes of evolutionary history as they serve in the maximization of parental fitness^1^. Reproduction requires resources that can be limited by several external and/or internal factors which determine the total reproductive effort^2^. Females invest a portion of their resources in their offspring (i.e., reproductive effort), but are faced with a trade-off: should the female use her reproductive resources to produce a large number of smaller offspring (r-selection) or a small number of larger offspring (K-selection). Recognition of trade-offs between these two extreme fecundity outcomes are well documented in terrestrial ecology^3,4^. Most terrestrial studies refer to the conceptual balanced model proposed by Smith and Fretwell^5^ based on two intuitive assumptions: (1) the existence of a trade-off between offspring number and size that means when energy invested in individual offspring increases (resulting in larger offspring) the total number of offspring decreases; and (2) a positive relationship between individual offspring size and its fitness. This model has proven to be more applicable to species that produce numerous small offspring without parental care which is a hallmark of r-strategists. Other studies confirmed that climate conditions could affect reproductive traits and female body size in ectotherms^6^. In the aquatic environment, the reproductive trade-offs among copepod species were mainly studied in ectoparasites^7,8^. In the literature, only a few examples dealt with zooplankton with a small number of papers concerned with copepods in particular. The trade-off between egg size and reproductive output (fecundity or reproductive effort) was analysed for a combination of copepod groups (free-living and parasitic taxa) and revealed a negative relationship^9,10^ following the Smith & Fretwell model. The only study dealing with a single species was made by Timi et al.^7^, showing no evidence of trade-off between fecundity and egg volume in the parasitic copepod *Lernanthropus cynoscicola*. However, the absence of trade-offs in this parasitic copepod could be due to the high resource availability that allowed a good allocation of energy to both egg size and number^7^. Most free-living copepods that are playing key roles in aquatic food webs face resource limitations as well as fluctuations of environmental factors, such as temperature, salinity and food^11,12^. Therefore, a trade-off between egg size and number could be expected. Empirical observations showed that individual egg carbon content could be modelled as a general power law (i.e., linear in a log–log plot) of egg diameter among several species of copepods^13^. The consideration of carbon content is common in copepod ecology because one of the key reproductive parameters expressing the reproductive effort is the specific egg production rate calculated as the proportion of female body mass (usually expressed in terms of carbon content) invested in the clutch mass (in terms of total egg carbon) per day.

The trade-off between egg and clutch size in copepods has received less attention and the possible reasons of switching between an r- and K-strategy by copepod females is not known as yet. Food quality and quantity is one parameter that was demonstrated to affect the trade-off between offspring number and egg size in the marine copepod *Euterpina acutifrons*^14^. Egg size in copepods was also demonstrated to have seasonal variability. Liang and Uye^15^ showed a clear seasonal pattern in egg diameter of the egg bearing copepod *Pseudodiaptomus marinus* with larger eggs produced by bigger females seasonally in winter and spring. Cavaleiro and Santos^8^ found a similar seasonality in the ectoparasitic copepod *Octopicola superba*. Generally, copepods are fast growing organisms and their reproductive output is often considered as an estimator of the quality of their environment. Environmental fluctuations such as in a pulsed resource system are otherwise known to have pronounced fitness consequences^16^. But no deep experimental study was devoted to test the ecological and evolutionary significance of the trade-off occurrence between clutch size and egg size in copepods as yet. Although clutch size and egg size are not the only reproductive traits affecting the fitness of copepod species, it is important to test their potential to switch between K and r strategies. In this study, based on a large database obtained under controlled conditions, we aimed to test the hypothesis of existence of a trade-off between clutch size and egg size.

In temperate ecosystems, copepods exhibit a clear seasonality in their life history traits with high reproductive activity during spring^12,15,17^. Several calanoid copepods as well as other crustacean zooplankter can modify their reproductive strategy by producing subitaneous eggs and diapausing eggs during unfavorable seasons^18–21^. From an ecological point of view, copepods could display a particular life history strategy with regard to offspring size and number. In addition to the empirical evidence of seasonal variability of egg size and egg number in a marine copepod^14,15^, the advantage of producing large eggs delivering offspring of high fitness was confirmed in a freshwater copepod *Cyclops kolensis*^22^. To the contrary, copepod females that adopt r-strategy could maximize their recruitment potential under favorable though unpredictable conditions. Therefore, an examination of the reproductive strategy of copepod females with respect to offspring size and number could improve the understanding of life cycle variations and adaptive reproductive evolutionary strategies of copepods at different environmental conditions^23^.

In comparison to free spawning copepods, clutch-bearing copepods use a reproductive strategy that likely results in lower egg mortality because eggs are carried by the female^24^. In addition, clutch-bearing copepods offer an advantage to test above trade-off theory because ovigerous females can have their body size (maternal control) and reproductive effort (including clutch and egg sizes) measured^17^.

The clutch-bearing copepod used in this study is the estuarine copepod *Eurytemora affinis*. It is a calanoid species that dominates, at times, the copepod communities in several estuaries and other aquatic ecosystems in the northern hemisphere^25,26^. In macrotidal estuaries, *E. affinis* evolved adaptations to keep the bulk of its population in the low salinity zone and to be exposed to high variability of both salinity and temperature^27^. Several field and laboratory studies showed that the clutch size of *E. affinis* is highly variable and could be affected by food, temperature and salinity^28–32^. Recently, Souissi and Souissi^17^ showed that some climatic anomalies that occurred during the critical period of *E. affinis* development (late winter-early spring) was detected when using simultaneously the average data of prosome length and clutch size. These negative events occurring in the field decreased significantly the reproductive effort expressed as clutch-size^17^. However, the above study did not explore the partition of the reproductive effort between clutch size and egg size.

In this study, we combined results obtained from two long-term experiments carried out simultaneously, following a multigeneration protocol to test our hypothesis. Both studies used the copepod *Eurytemora affinis* from the Seine estuary which was cultured throughout several generations in the laboratory. The first study aimed to test the effect of a warming scenario of 4 °C on a warm acclimated population of *E. affinis* (20 °C) using two salinities^33^. Whereas the second study investigated whether some morphological traits (e.g., female size, egg size) obtained in a cold acclimated population of *E. affinis* (7 °C) could be selected after severe heat-shock, similar to a heat-shock wave^34^ with a direct transfer of the population to 24 °C^35^.

Starting from the same batch culture of *E. affinis* individuals isolated from the field we derived progressively three population lines, one acclimated to late winter early spring like temperature (cold = 7 °C) and another one acclimated to summer like temperature (warm = 20 °C). Both populations were maintained at optimal salinity of 15 PSU whereas the third population line combined warm (20 °C) and higher and stressful salinity (25 PSU). The last experimental condition corresponded to the combination of low river discharges only observed during summer leading to more marine water intrusion to the *E. affinis* habitat. These initial conditions observed during several generations allowed to test under controlled conditions the effects of three critical situations that the population encounters in the Seine estuary. Then the three population lines were transferred to more stressful thermal conditions of 24 °C without changing the salinity and followed during 5 consecutive generations. This allows to have additional sets of data corresponding to a severe heat shock of the cold acclimated population and a warming scenario of + 4 °C applied only to summer conditions for the two other population lines. Having these data at 24 °C will allow to test the sensitivity of any reproductive strategy observed in the initial conditions to higher and stressful temperature. We focused mainly on the morphology and the reproductive strategy of *E. affinis* and made the hypothesis that the reproductive strategy of this population should show a trade-off between clutch and egg size under different thermal and haline conditions. We intended to investigate; first, whether an acclimation of a copepod culture to certain conditions prior to the initiation of experimental treatments matters; and whether temperature and/or salinity increase could affect the total reproductive effort of *E. affinis*. Our main hypothesis was that the long-term acclimation of copepods to initial conditions (7 or 20 °C) can generate a trade-off strategy whereas the warmer final conditions (24°) could be very stressful to the development of *E. affinis* and consequently will not show any trade-off pattern. We also tested whether the selection regime may have had an effect in the trade-off and whether it can be observed within the same cohort (i.e., generation).

## Material and method

### Experimental conditions

**Figure 1:**
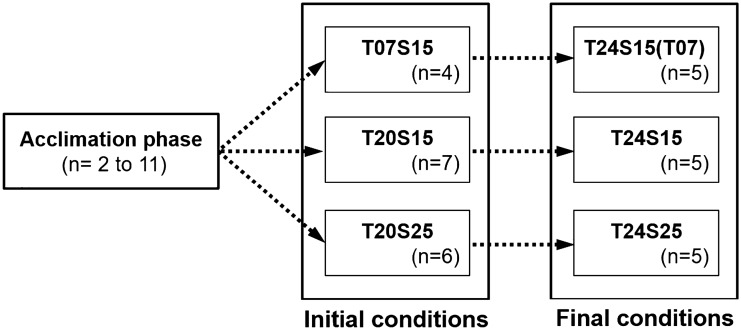
Flowchart of experimental procedures. In the left box, n indicates the number of generations under acclimation phase (n = 2 for T = 7 °C and n = 10 or 11 for T = 20 °C). The two larger boxes correspond to the initial (left) and final (right) conditions. For each condition TXSY indicated that temperature was set to X °C and salinity to Y PSU. Two conditions were realized at T = 24 °C and S = 15PSU, to make a distinction the heat shock treatment was designed T24S15(07). In both boxes n indicates the number of generations followed at each condition.

An *E. affinis* population was sampled from the Seine estuary (France) in the low salinity zone under the Tancarville bridge (49°26ʹN–00°16ʹW) at a water temperature of 12 °C and a salinity of 5 PSU. Copepods were then maintained in the laboratory in a 25 L glass aquarium at temperature 15 °C and salinity 15 PSU during two generations. Afterwards, the batch culture was split into two aquaria (25 L) and a progressive acclimation to cold (7 °C) and warm (20 °C) temperatures was realized, keeping the salinity at 15 PSU. Then a multigeneration study of the cultures follow up of the culture was launched in 2 L beakers using 40 ovigerous females sorted from each of the batch cultures by using the same protocol^33^. The first beaker culture was conducted at 7 °C and 15 PSU (T7S15) and the second one was kept at elevated 20 °C and 15 PSU (T20S15). After one generation at 20 °C and salinity 15 PSU, 40 ovigerous females sorted randomly were used to initiate the second generation of the population T20S15 and 40 additional ovigerous females were sorted randomly to derive a third initial condition, corresponding to the population T20S25 where only salinity was increased. The selected cold and warm temperature corresponded to the critical period of the development of *E. affinis* population in the Seine estuary (early spring and late summer). Whereas the use of higher salinity can occur only during the warmer season when the river discharges are very low^27^. These three experiments were followed for at least 2 generations for an acclimation period, before testing the effects of temperature increase. The acclimation lasted for 2 generations at 7 °C and and for 10 to 11 generations at 20 °C (Fig. 1). The acclimation period at 7 °C compared to 20 °C was selected as comparatively short due to the length of the generation time. The mean copepod developmental time was 42 days at 7 °C and 12 days at 20 °C.

After the acclimation phase, 4 successive generations were carried out under T7S15, 7 generations under T20S15 and 6 generations under T20S25 (Fig. 1). The experimental protocol of the generation’s follow-up was detailed in Souissi et al.^33,35,36^. In the present study, each generation was considered as a replicate (or pseudo-replicate) of the experimental condition. Furthermore, for some statistical analyses all individual data for each ovigerous female were also used (see Supplementary Information). All initial conditions (T7S15, T20S15 and T20S25) were then transferred to a higher temperature of 24 °C and followed during 5 subsequent generations (Fig. 1). During the whole period of the experiment (~ 1 year) copepods were fed daily ad libitum with *Rhodomonas marina.*

Between 20 to 40 ovigerous females were sorted from each generation sample to measure all morphological and reproductive traits. Each sorted female was observed under an inverted microscope. Then the prosome length and width were measured as detailed in Souissi et al.^36^. Afterwards, the clutch size was counted by tearing the clutch carefully and individual measurements of 5 to 15 eggs per clutch were done to determine the egg size. All the size measurements were conducted using Image J software^35^.

### Selection of reproductive traits

The fecundity (F = number of eggs female^-1^) of *E. affinis* was estimated as the number of clutches multiplied by the clutch sizes. In this study only the first clutch produced by *E. affinis* females was considered. Two variables were used to estimate egg size: egg diameter (ED) and egg volume (EV). Egg volume was calculated based on their spherical shape. The total reproductive effort was then defined as the total volume of the clutch (CV, CV = clutch size x mean EV) because reproductive traits in copepods are usually correlated to female body size. The prosome length (PL) and the prosome volume (PV) were used as proxies of female size. All measured traits obtained in this study were given as means per generation.

### Statistical analyses

We used Pearson’s correlation coefficient to test a possible relationship between all variables measured using original and log-transformed data. All correlations with log-transformed variables gave lower *r*-values than the original data. Therefore, only linear relationships were selected. Because the reproductive output of copepods is often determined by female body size, we first verified this statement. The correlations between reproductive trait and female body size were very similar, when either PL or PV was used. As a consequence, only relationships with female PL, which is the commonly used variable to describe body size in copepods, were retained.

Because simple linear regressions between clutch size, egg size, and total reproductive effort (dependent variables) and female PL (independent variable) provided the most significant relationships (compared to multiple linear regressions), we decided to perform a simple residual analysis to test the existence of trade-offs between egg size and number. A particular interest was given to the relative relationship between ED and clutch size (CS), after removing the effects of female body size. All statistical analyses were done with Matlab software (Mathworks, Inc., Portola Valley, CA, USA).

In order to assess the robustness of the linear relationship between residuals, the input data (x and y) were resampled using a bootstrap technique available from Matlab Software. This generated 10,000 resampled values of the correlation coefficient (*r*) and the slope (a) of the regression function: y = a*x. Then the distribution of the simulated values for each parameter was represented by a histogram.

In order to compare the three key variables, prosome length, clutch size, and egg size at initial conditions (T07S15, T20S15 and T20S25) and final conditions after transfer to 24 °C (T24S15(T07), T24S15, T24S25), a non-parametric ANOVA (Kuskal-Wallis) was realised. Then, for each selected parameter, the three pairs composed of initial condition and final condition after transfer at 24 °C, were tested by a two-sided Wilcoxon rank sum test.

Our experiment contains 4 factors that can be summarised as follow:

i. The acclimation/selection (if any) regime that provides 3 different populations T7S15, T20S15 and T20S25. We coded the data following three populations/lines corresponding to the 3 initial conditions as well as the generations followed after transfer to 24 °C.
ii. The generation number was used as a second factor within each experimental condition (3 initial and 3 final conditions, providing a total of 6 treatments).
iii. Temperature: 7 °C, 20 °C and 24 °C
iv. Salinity: 15 PSU and 25 PSU

After this preparation of the data base we used n-way ANOVA (function anovan under Matlab) with interaction to test the significance of each factor for each reproductive trait selected in our study: Prosome length (PL), clutch size (CS) and egg diameter (ED).

In addition to the full n-way ANOVA with the 4 factors mentioned above, we also performed two additional 3-way ANOVA by considering in each analysis Temperature and Salinity plus factor1 or factor2 (population line or generation). Because the statistical conclusions did not change when only 3 factors were tested, we kept the full factors ANOVA in the subsequent analyses.

## Results

**Figure 2:**
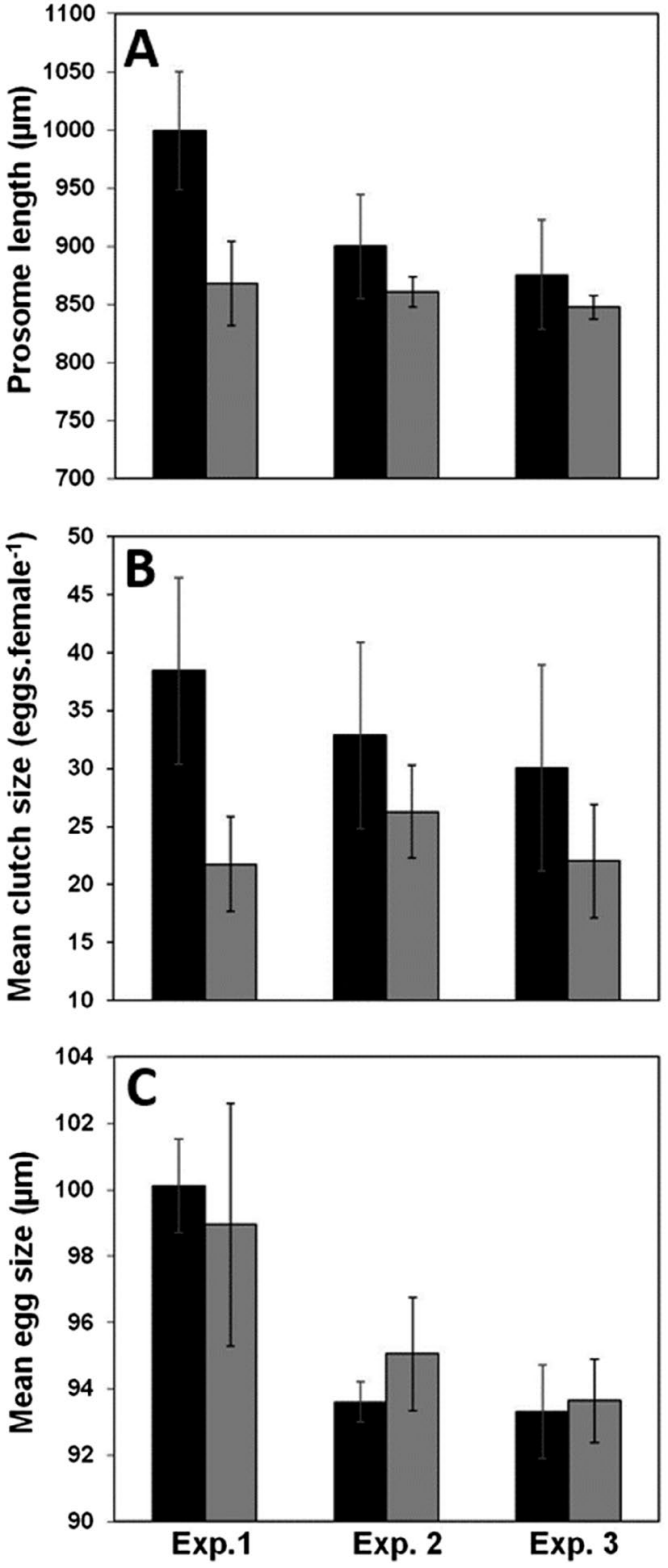
Each bar represents the mean of generations under different conditions. Exp. 1: experiment conducted under T07S15 then T24S15(T07); Exp.2: experiment conducted under T20S15 then T24S15; Exp.3: experiment conducted under T20S25 then T24S25, Black bars: initial conditions, grey bars: final conditions (transfer to 24 °C).

Figure 2 shows the variability of the mean values of the three reproductive traits PL, CS and ED at all experimental conditions. At salinity 15 PSU, temperature increased from 7 to 20 °C reduced female PL by almost 10% from 999.14 µm to 899.82 µm (Fig. 2A). At 20 °C the salinity increase reduced slightly (2.72%) PL to 875.38 µm (Fig. 2A). For PL, a significant difference between three experiments was observed at initial conditions (Fig. 2A, p = 0.020) but not at final conditions (Fig. 2A, p = 0.406). In fact, at 24 °C, differences between the mean values of PL were very low with a decreasing trend from 867.78 µm (Exp. 1) to 860.33 µm (Exp. 2) and to 847.48 µm (Exp. 3). The comparison between initial and final conditions was only significant between the extreme temperatures 7 °C and 24 °C (Fig. 2A, p = 0.029) where a decrease in mean PL was about 13.15%. On the contrary, for warm acclimated conditions the decrease of PL after transfer to 24 °C was 4.39% and 3.19% in Exp. 2 and Exp. 3, respectively (Fig. 2A).

The CS was the most variable reproductive trait. No significant differences within initial as well as final conditions were observed here (Fig. 2B, p > 0.05). As for prosome length, the only significant difference between initial and final conditions was for the extreme temperatures 7 °C (38.42 eggs/female) and 24 °C (21.75 eggs/female). To the contrary a decrease in CS was relatively less pronounced for warm acclimated populations (Exp.2 & Exp.3, Fig. 2B). At salinity 15 PSU, a temperature increase by 4 °C induced a decrease in fecundity by 19.95% from 32.84 eggs/female to 26.29 eggs/female (Fig. 2B). For the stressful salinity of 25 PSU the CS decrease was around 26.64% from 30.05 eggs/female at 20 °C to 22.04 eggs/female at 24 °C (Fig. 2B).

PL and CS showed similar patterns of variability. However, for egg size (ED) the pattern was different, because a Kruskal–Wallis test was significant for both initial (p = 0.013) and final (p = 0.024) conditions. This was certainly due to the high values of egg size at the lowest temperature of 7 °C (100.12 µm, Fig. 2C) that remained high even after transfer to 24 °C (98.95 µm, Fig. 2C). To the contrary, no significant difference was observed between the initial and final condition within a single experiment (p > 0.05). For warm acclimated populations the mean values of ED were 93.59 µm, 93.31 µm, 95.05 µm and 93.64 µm for the 4 experimental conditions T20S15, T20S25, T24S15, and T24S25, respectively.

For PL and CS, the n-way ANOVA results were similar. Only the factors generation, and temperature were significant (p < 0.01). The interaction ‘generation x temperature’ was also significant but the significance level was higher for PL (p = 0.007) compared to CS (p = 0.0346). For CS the interaction generation x salinity was not significant but the p-value was low (p = 0.0969). To the contrary the n-way ANOVA did not show any significant factor for ED with the lowest p-values observed for the interaction population x generation (p = 0.0713) and the interaction generation x salinity (0.0731).

**Figure 3:**
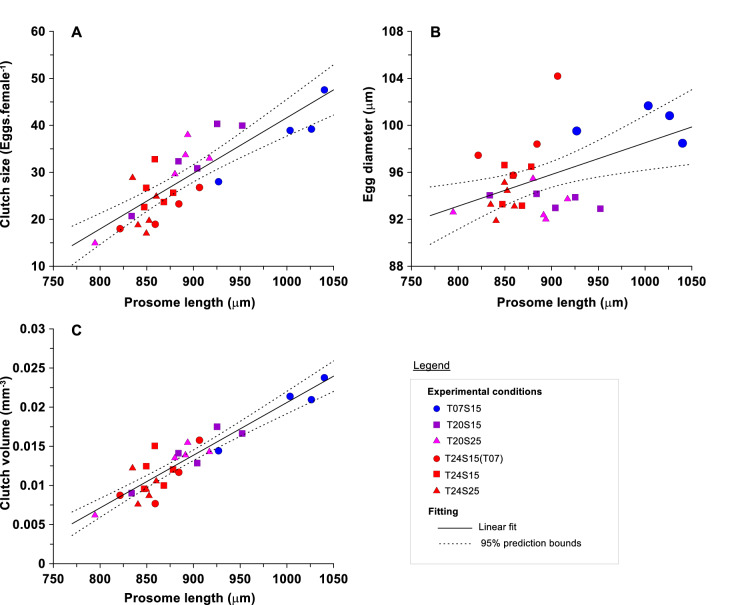
Relationship between *E. affinis* female prosome length with clutch size (**A**), egg diameter (**B**) and clutch volume (**C**). The rectangles contain the labels of each population used from laboratory experiments as shown in the flow chart of Fig. 1. The solid line is the regression line and the dashed lines indicate the 95% confidence interval. The results of the regression analyses were indicated in Table 1.

The mean PL decreased as temperature and salinity increased (Table 1) and the largest mean PL was obtained at the lowest temperature. Female PL explained 85.1%, 70.0% and 25.6% of the total variance in CV, CS and ED, respectively (Table 1). The relatively low *r*^2^ obtained between ED and PL was due to the high dispersion of the data observed in all generations (Fig. 3B). ED showed no significant relationship with CS (*r*^2^ = 0.032, *p* = 0.362). CS (Fig. 3A) and CV (Fig. 3C) showed a significant positive trend with PL.

**Table 1:** Results of the linear regression analysis (Y = aX + b) between reproductive traits of *E. affinis* as dependent variables (CS, ED and CV) and PL (independent variable) as well as the relationship between the residuals from these regressions (CS vs PL) and (ES vs PL).

	Y = aX + b		a (slope)	b (intercept)	n	R^2^	Adj-R^2^	p
All conditions	Y = CS	X = PL	0.118 ± 0.031	− 76.806 ± 27.835	28	0.700	0.689	< 0.0001
	Y = ED	X = PL	0.027 ± 0.019	71.548 ± 16.483	28	0.256	0.228	< 0.01
	Y = CV	X = PL	6.7 10^–5^ ± 1.14 10^–5^	− 0.047 ± 0.010	28	0.851	0.845	< 0.0001
	Y = Resid. (CS vs PL)	X = Resid (ES vs PL)	− 0.874 ± 0.582	0.014 ± 1.551	28	0.268	0.240	< 0.01
Initial conditions	Y = CS	X = PL	0.105 ± 0.040	− 62.889 ± 36.696	14	0.733	0.710	< 0.0001
	Y = ED	X = PL	0.034 ± 0.021	63.733 ± 19.510	14	0.511	0.470	< 0.01
	Y = CV	X = PL	6.5 10^–5^ ± 1.08 10^–5^	− 0.045 ± 0.010	14	0.935	0.930	< 0.0001
	Y = Resid. (CS vs PL)	X = Resid (ES vs PL)	− 1.502 ± 0.712	− 0.041 ± 1.590	14	0.638	0.607	< 0.001
Final conditions	Y = CS	X = PL	0.068 ± 0.126	− 34.599 ± 108.290	14	0.102	0.027	0.265
	Y = ED	X = PL	0.091 ± 0.069	17.190 ± 59.521	14	0.408	0.358	0.014
	Y = CV	X = PL	6.5 10^–5^ ± 6.05 10^–5^	− 0.045 ± 0.052	14	0.316	0.259	0.037
	Y = Resid. (CS vs PL)	X = Resid (ES vs PL)	− 0.085 ± 1.143	− 0.008 ± 2.616	14	0.002	< 0.0001	0.875

*CS* Clutch size, *ED* Egg diameter, *CV* Clutch volume, *PL* Prosome length, *ES* Egg size, *Resid* Residual.

**Figure 4:**
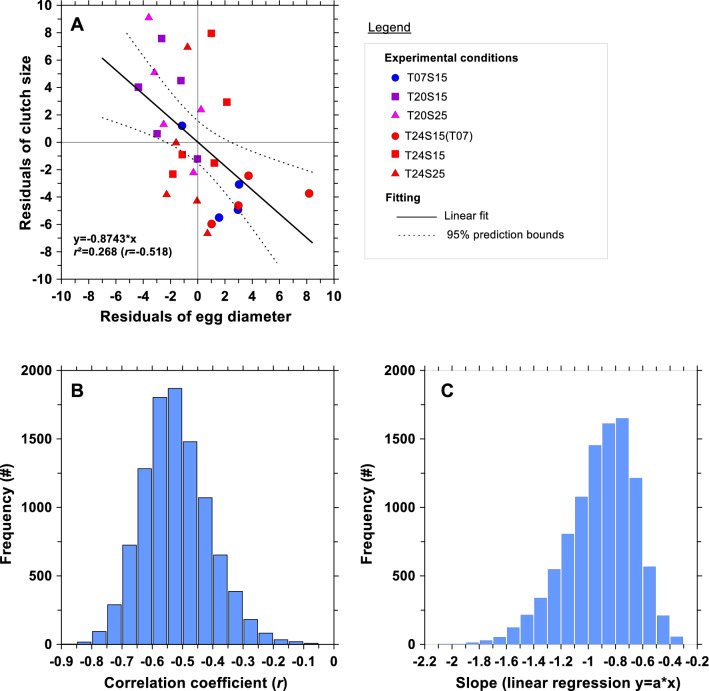
(**A**) Relationship between residuals of linear regressions of mean egg diameter and clutch size against female prosome length. The solid line is the regression line and the dashed lines are the 95% confidence interval. The grey lines intersect with the origin (0,0). The legend shows the labels of each population that are the same as in Fig. 3. (**B**) Histogram of the distribution of the 10,000 values of the coefficient of correlation (*r*) generated by the resampling bootstrap method. (**C**) Histogram of the distribution of the 10,000 values of the slope parameter of the linear regression y = a*x.

We regressed the residuals of the linear regressions of both CS and ED versus PL which resulted in a negative relationship (Fig. 4A). This linear regression explained almost 27% of the total variance in CS using ED as a predictor (Table 1). This negative relationship between residuals suggests that for a given PL, females with small CS (i.e., symbols below the regression line in Fig. 3A) tend to produce larger eggs (i.e., symbols above the regression line in Fig. 3B). The reverse was also suggested, where females with larger CS tend to produce smaller eggs (Fig. 3A,B). The data from the populations initially acclimated to the low temperature (7 °C) were situated in the same area of the graph that corresponded to relatively smaller CS and larger ED (Fig. 4A). In contrast, populations reared at 20 °C (both salinities) were situated in an opposite area of the graph, an area that corresponded to relatively larger CS and smaller ED (Fig. 4A).

After transfer to 24 °C, the ED of these populations did not appear to change (i.e., residuals of ED *vs* PL were close to 0) compared to other temperatures but did show high variability in CS (CS remained smaller than in the other treatments). We separated the initial and final experimental conditions and performed the same regression between the residuals of CS and ED. The residual analysis was highly significant for the initial conditions (*r*^2^ = 0.638, n = 14, *p* < 0.001). However, the relationship was not significant after the experiment was run (*r*^2^ = 0.002, n = 14, *p* = 0.875). This suggests that the negative slope observed in Fig. 4A was driven primarily by the initial conditions, which suggest for a trade-off. However, when conditions became stressful, no trade-off was observed (Table 1). The resampling of the data by bootstrap confirmed that the linear relationship obtained in Fig. 4A was very robust as shown by the distribution of the coefficient of correlation with very significant negative values (Fig. 4B) and negative slopes (Fig. 4C).

Additional analyses using individual data (each ovigerous female) confirmed that the trade-off between clutch size and egg size can be observed within a generation (see Stable 1). In fact, the trade-off was statistically significant in 13 different generations among all observed generations (n = 35). When individual data were used the robustness of the trade-off in initial conditions (SFig. 1A) was much higher than the one observed after transfer to 24 °C (SFig. 1D).

## Discussion

In this study a large data-base of the main reproductive traits of the estuarine copepod *E. affinis* (female size, clutch size and egg size) was compiled from a long-term experiment conducted at 3 temperatures (7 °C, 20 °C and 24 °C) and two salinities (15 PSU and 25 PSU). Our results confirmed that temperature was the main factor that explained the variability of the reproductive traits studied here. The hypothesis of the existence of a trade-off between clutch size and egg size was confirmed whatever the data was averaged for each generation or considered individually for each female. The trade-off can also be observed within the same generation but not systematically. The trade-off pattern was mainly driven by the initial conditions where the populations were acclimated to three critical situations of temperature and salinity that can be observed in the field. The transfer of populations to 24 °C engendered a stress to the population without strong trade-off pattern. After positioning our results with the available literature the ecological implications of our findings were discussed.

Our study applied a multi-generational approach to determine whether a trade-off existed between clutch size and egg diameter for *E. affinis*. The experiments conducted in this study were performed on a single population of *E. affinis* that originated from the Seine estuary and aimed to test a global warming scenario that can lead to an increase in both temperature and salinity^33,40^.

The measure of clutch size, commonly used in the literature^13,28^, was linearly related to female PL (Fig. 3A). In contrast to Ban^28^ who found a power relationship between PL and CS in *E. affinis*, our fit was linear. These differences could be due to experimental differences between the two studies. Ban^28^ focused on the effect of food concentration on reproductive output for a single isolate of copepods, while our protocol was based on a batch culture and multigenerational observations. In both cases, a general positive trend between CS and PL was observed, demonstrating higher fecundity in larger females. When the total reproductive effort was calculated (the product of CS and mean EV (that is CV)), its relationship with female PL was also positive (see Fig. 3C and Table 1) as a direct consequence to EV changes. In fact, if the EV was held constant, the relationship between either CS or CV (CS x EV) *versus* PL resulted in similar regression coefficients and slopes (Table 1).

We found that the mean egg diameter of *E. affinis* can vary from 91.96 μm to 104.19 μm, with an overall increase of 13.30% and 45.44% in ED and EV, respectively. The variability of ED was mainly explained by female size and less so by temperature and salinity (see Fig. 3B). The positive relationship between PL and ED was present in all combined and separate conditions (see Table 1). This result suggests that *E. affinis* can adjust both the size and the number of offspring. In order to remove the effect of female size on reproductive traits, we used the residuals from regression with PL and our results revealed that the presence of a trade-off between egg size and egg number existed. A significant negative trend was observed between residuals (see Fig. 4A and Table 1), confirming that the allocation of the reproductive effort to egg number and egg size was not independent. The negative trend of Fig. 4A (and SFig. 1 when the analysis was performed with individual data) is explained by the fact that *E. affinis* females were investing more energy in their offspring (i.e., at low temperature 7 °C) producing relatively smaller clutches, whereas ovigerous females with relatively bigger CS (i.e., at 20 °C) invested less in their individual offspring which resulted in smaller eggs. To our knowledge, this study provides the first experimental confirmation of the presence of a trade-off in the reproductive strategy of a clutch-bearing copepod.

The phenomenon of a trade-off between egg number and egg size was earlier observed in freshwater cladocerans^41^ as well as in terrestrial invertebrates^42^. Only one detailed study at the species level exists for copepods and this species is the parasitic *Lernanthropus cynoscicola*^7^. Thus our ability to complete a comparative analysis for pelagic copepods is limited. At higher taxonomic levels, Caley et al.^10^ suggested that total reproductive effort (RE) and egg size in copepods did not evolve independently. The high heterogeneity in the literature values and the high variability in the residuals (see Fig. 1 in Caley et al. 2001) resulted in a low amount of variability of egg size (< 11%) which is explained by RE. Caley et al.^10^ recognizing the limitations of using an estimation of the total reproductive effort that was not independent from the estimation of the egg size and suggested that future studies should focus on the simultaneous allocation decisions rather than on sequential ones as well as other genetic or selective mechanisms. We confirmed their conclusions at the specific level by using *E. affinis* as a biological model. The observed trade-off between egg size and egg number was mainly driven by the initial conditions as confirmed by our analyses either using mean data per generation (Table 1) or individual data (SFig. 1). It is possible that this finding resulted from a selection process where individuals experiencing the same conditions during multiple generations produce the same trade-off. However, the test of the existence of a trade-off for each generation did not show any clear selective breeding pattern of this mechanism (see Stable 1). We showed that after the temperature increased to 24 °C (due to a possible warming or to an artificial heat shock) the trade-off between egg size and egg number observed in the initial conditions disappeared (or was very weak when individual data were used, SFig. 1D). This means that either the higher temperature of 24 °C disrupted the reproductive trade-off because it was very stressful or that the population required a much longer period to acclimate to this new environmental temperature. Whatever the exact mechanisms causing the absence of a reproductive trade-off at higher temperature, future studies on the possible effects of temperature increase on the life cycle traits should consider the inter-dependence of reproductive traits (see Beyrend-Dur et al.^43^).

The experimental temperatures used in the initial conditions (7 °C and 20 °C) correspond to the observed extremes in the low salinity zone of the Seine estuary during the seasonal growth of the population of *E. affinis* between late winter and early summer^27,44^. Consequently, *E. affinis* should optimize its reproductive strategy at both temperatures to ensure optimal development of its offspring. At lower temperature (7 °C), *E. affinis* females grew slowly but showed the lowest mortalities among larger sized individuals^33^. The positive relationship between female size and fecundity was shown for several ectotherms, including the copepod *E. affinis*^17,28,33^. Our study confirmed that both fecundity and the quality of individual offspring was enhanced with larger female size. The upper thermal limit (20 °C) corresponded to a decline of the *E. affinis* population in the Seine estuary^45^. At this temperature under laboratory controlled conditions, the trade-off between egg size and number favored the production of smaller-sized eggs. In the Seine estuary, the *E. affinis* population faces its highest mortalities and increased predation due to high temperatures in summer^46–48^. A short-term shift in reproductive strategy could improve the number of individuals that survive in this unfavorable season. Moreover, we showed that the development of *E. affinis* is possible at 20 °C in the laboratory when all other external factors (i.e., food, predation, competition) were removed. Even when temperature was raised to 24 °C (including the artificial heat shock treatment from 7 °C to 24 °C), all populations were able to survive and reproduce without showing any apparent trade-off pattern. A detailed examination of the data (Fig. 4A) confirmed that the population originating from 7 °C kept the same initial trade-off after transfer to 24 °C. This suggests that the initial acclimation to 7 °C selected females adopting a strategy that favored the investment in individual offspring, even after 5 generations at 24 °C. On the other hand, a 4 °C increase in the other treatments for copepods acclimated to the upper thermal limit (20 °C) seemed to disturb the trade-off strategy during five subsequent generations. The summer population of *E. affinis* may face such an increase in temperature and their response to this challenge may be impacted significantly according to our results.

We confirmed in this study that ovigerous females of *E. affinis* appear capable of adjusting their egg size in addition to their clutch size. The variability of these two reproductive traits was highly correlated to female body size. However, when the effect of body size was removed, we found that females appeared to display a clear trade-off between size and number of offspring, a trend that also depended upon environmental conditions. This study is the first experimental test of Smith and Fretwell’s^5^ model of a clutch-bearing copepod. Our data support this model for the copepod *E. affinis*. As in the case of Caley et al.^10^, we demonstrated that the allocation of reproductive energy was not simply sequential but rather simultaneous. In fact, most theoretical studies in this field have modeled the evolution of total allocation to offspring and the subsequent division of this allocation into many small versus few large offspring as independent. Our study suggested that these life-history traits may be evolutionarily linked. This strategy offers substantial ecological advantages. In temperate ecosystems, the transition from winter to spring seasons is crucial in determining the strength of the maximum density of *Eurytemora* like species^17,27^. Consequently, reproducing in late winter and early spring, the most critical seasons^17^ large eggs will enhance the survival of the offspring. This can ensure a high recruitment and a maximum density of the *E. affinis* population that occurs regularly in May–June in the Seine estuary. However, during the warmer season the physiological performance of *E. affinis* is reduced compared to free spawning species, such as in the genus *Acartia*^33^. In addition, the autumn season and particularly the month of November seems to be a critical month in the condition of *E. affinis* in Europe^17^. Hence, investing in offspring quality during summer where the mortality is highest also due to predation^47,49^ would not be advantageous for this species. Rather, switching to an r-strategy by producing more eggs with smaller size could lead to a reduced recruitment of pre-winter generations, that are capable of maintaining a sufficient overwintering adult stock. The life expectancy of *E. affinis* females can reach 2 months at 10 °C^29^ and certainly more at much lower temperature such as 6–8 °C. This means that the reproduction of *E. affinis* is continuous in the Seine estuary with a high variability of size: with generally larger females during the colder season and smaller females during the warmer season^17^. The trade-off between CS and ED was also observed at a single generation level (one cohort) at all experimental conditions without showing any effect due to a possible selective pressure (see Stable 1). But when individual data were combined in a single analysis the trade-off between CS and ED was clearly much stronger in initial conditions rather than final and warmer conditions (see SFig. 1). This confirms the high reproductive plasticity of this species adapted to live in a highly variable environment and opens a new perspective to assess in future studies the role of such reproductive strategy in the ecological performances of this species or similar congeneric ones. Furthermore, *E. affinis* in the Seine estuary is capable of producing a small percentage of diapausing or quiescent eggs that may contribute to an adaptive trait in the evolutionary ecology of this species^21^.

Previous studies on the reproduction of the copepod *E. affinis* often focused on the fecundity^33^ or carbon content of the produced clutches^37^. However, some studies assumed that the egg size and its carbon content are constant^37^. For example, Hirche^38^ compared the reproductive strategies of *E. affinis* to the broadcast spawner *Acartia tonsa* using a fixed ED for both species (82 μm and 73 μm, respectively). This approach is obviously flawed in light of the observed variability in egg size that reflects a possible change in the reproductive investment of the female. For example, Crawford and Daborn^39^ showed that the egg diameter of *Eurytemora herdmani* (congeneric species of *E. affinis*) inhabiting a turbid estuary varied between 82 and 99 μm. Despite only 4 measurements of ED (see Table 1 in Crawford and Daborn^39^), a reanalysis of their data confirmed a significant negative correlation between CS and ED (*r* = 0.94, n = 4, p < 0.05), suggesting the possible existence of a trade-off between these two reproductive traits. Therefore, it is possible that *E. affinis* populations exhibit such a trade-off and our results strongly support this hypothesis.

The extrapolation of our results in an ecological context appears to be related to the observed seasonality of the *E. affinis* population in the Seine estuary. *E. affinis* appears as an assemblage of cryptic species composed of different populations inhabiting fragmented habitats in the northern hemisphere^50^. Consideration of its inter-population variability can lead us to a better understanding of its varied reproductive strategies. In conclusion, the present study suggests that reproductive strategies of *E. affinis* can be applied to optimal reproductive theory.

## Supplementary Information

Supplementary Information.
