# Supplementary material for: Reproductive trade-offs of the estuarine copepod Eurytemora affinis under different thermal and haline regimes
Source: Sci Rep. 2021 Oct 11;11:20139. doi: 10.1038/s41598-021-99703-0 (PMC8505402; doi:10.1038/s41598-021-99703-0)
Supplement: Supplementary file 1 — Supplementary Information. [file 41598_2021_99703_MOESM1_ESM.docx]

SUPPLEMENTARY MATERIAL

**Reproductive trade-offs of the estuarine copepod *Eurytemora affinis* under different thermal and haline regimes**

Anissa Souissi^1,2^, Jiang-Shiou Hwang^3,4,5**^, Sami Souissi^1*^

**^1^** Université de Lille, CNRS, Université du Littoral Côte d’Opale, UMR 8187 LOG, Laboratoire d’Océanologie et de Géosciences, Station Marine de Wimereux, F-59000 Lille, France.

**^2^** Univ. Littoral Côte d'Opale, UMR 1158 BioEcoAgro, TERRA Viollette, USC Anses, INRAe, Univ. Lille, Univ. Artois, Univ. Picardie Jules Verne, Univ. Liège, Yncréa, F-62200 Boulogne-sur-Mer, France

**^3^** Institute of Marine Biology, National Taiwan Ocean University, 202 Keelung, Taiwan

**^4^** Center of Excellence for Ocean Engineering, National Taiwan Ocean University, Keelung 20224, Taiwan

**^5^** Center of Excellence for the Oceans, National Taiwan Ocean University, Keelung 202, Taiwan

* Corresponding author: Sami Souissi. Tel: +33321992908, Fax: +33321992901,

e.mail: [sami.souissi@univ-lille.fr](mailto:sami.souissi@univ-lille.fr)

** Co-corresponding author: Jiang-Shiou Hwang. E-mail: Jshwang@mail.ntou.edu.tw

LIST OF MATERIAL

Supplementary STable 1.

Supplementary SFigure 1.

**STable 1.** Results of trade-off analyses performed for each generation and for all experimental conditions. The values of temperature (Temp.), salinity, the generation number and the number of females used in each generation (n females) are indicated in the first 4 columns. The slope (a) of the linear regression y=a*x between the contrasts of clutch size *vs* egg diameter (using the same methodology as in Figure 4A) and the coefficient of determination R² are indicated in columns 5 and 6. The last two columns (7 and 8) correspond to the outcome of the statistical test. The p-values below the threshold 0.05 corresponding to significant regressions are indicated in bold. The empty cells indicate that it was not possible to apply the trade-off statistical procedure and this mainly when the initial regression between CS and/or ED with PL was not significant.

| Temp. | Salinity | Generation | n females | slope | R² | F-stats | p-value |
| --- | --- | --- | --- | --- | --- | --- | --- |
| 20 | 15 | 10 | 10 | -2.40 | 0.654 | 15.150 | **0.00459** |
| 20 | 15 | 11 | 19 |  |  |  |  |
| 20 | 15 | 12 | 48 |  |  |  |  |
| 20 | 15 | 13 | 21 | -2.51 | 0.208 | 4.981 | **0.03787** |
| 20 | 15 | 14 | 40 | -1.32 | 0.217 | 10.551 | **0.00243** |
| 20 | 15 | 15 | 40 | -0.71 | 0.058 | 2.329 | 0.13530 |
| 20 | 15 | 16 | 40 | -0.19 | 0.064 | 2.616 | 0.11404 |
| 20 | 15 | 17 | 40 | 0.08 | 0.000 | 0.014 | 0.90800 |
| 20 | 25 | 9 | 10 | -2.40 | 0.654 | 15.150 | **0.00459** |
| 20 | 25 | 10 | 5 |  |  |  |  |
| 20 | 25 | 11 | 27 |  |  |  |  |
| 20 | 25 | 12 | 40 | -3.80 | 0.484 | 2.813 | 0.19209 |
| 20 | 25 | 13 | 40 | -2.13 | 0.319 | 17.790 | **0.00015** |
| 20 | 25 | 14 | 40 | -0.01 | 0.000 | 0.001 | 0.97937 |
| 20 | 25 | 15 | 23 | -0.45 | 0.043 | 0.947 | 0.34168 |
| 20 | 25 | 16 | 19 | -0.10 | 0.026 | 0.460 | 0.50663 |
| 7 | 15 | 3 | 40 | -2.22 | 0.473 | 9.867 | **0.00939** |
| 7 | 15 | 4 | 40 |  |  |  |  |
| 7 | 15 | 5 | 40 | -2.42 | 0.394 | 24.721 | **0.00001** |
| 7 | 15 | 6 | 40 | -0.12 | 0.001 | 0.052 | 0.82126 |
| 24 | 15 | 41 | 40 | -2.22 | 0.473 | 9.867 | **0.00939** |
| 24 | 15 | 42 | 40 |  |  |  |  |
| 24 | 15 | 43 | 58 | -1.65 | 0.207 | 14.614 | **0.00033** |
| 24 | 15 | 44 | 6 | 2.27 | 0.234 | 1.222 | 0.33099 |
| 24 | 15 | 45 | 22 | -0.79 | 0.035 | 0.724 | 0.40493 |
| 24 | 15 | 18 | 40 | -2.22 | 0.473 | 9.867 | **0.00939** |
| 24 | 15 | 19 | 40 |  |  |  |  |
| 24 | 15 | 20 | 40 | -2.42 | 0.394 | 24.721 | **0.00001** |
| 24 | 15 | 21 | 40 | -0.12 | 0.001 | 0.052 | 0.82126 |
| 24 | 15 | 22 | 40 | -0.27 | 0.020 | 0.763 | 0.38795 |
| 24 | 25 | 17 | 40 | -2.22 | 0.473 | 9.867 | **0.00939** |
| 24 | 25 | 18 | 40 |  |  |  |  |
| 24 | 25 | 19 | 16 | -2.45 | 0.189 | 3.265 | 0.09233 |
| 24 | 25 | 20 | 22 | -2.32 | 0.629 | 33.930 | **0.00001** |
| 24 | 25 | 21 | 40 | -0.10 | 0.001 | 0.039 | 0.84477 |

| 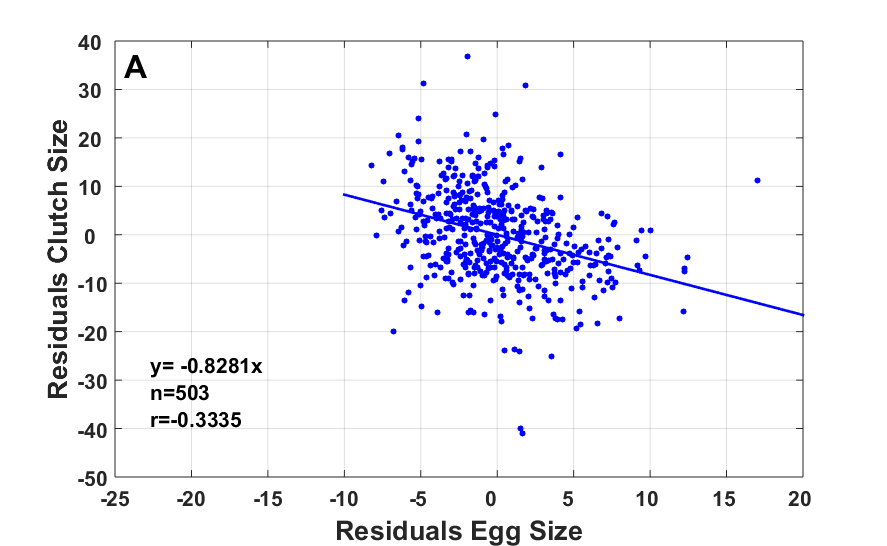 | 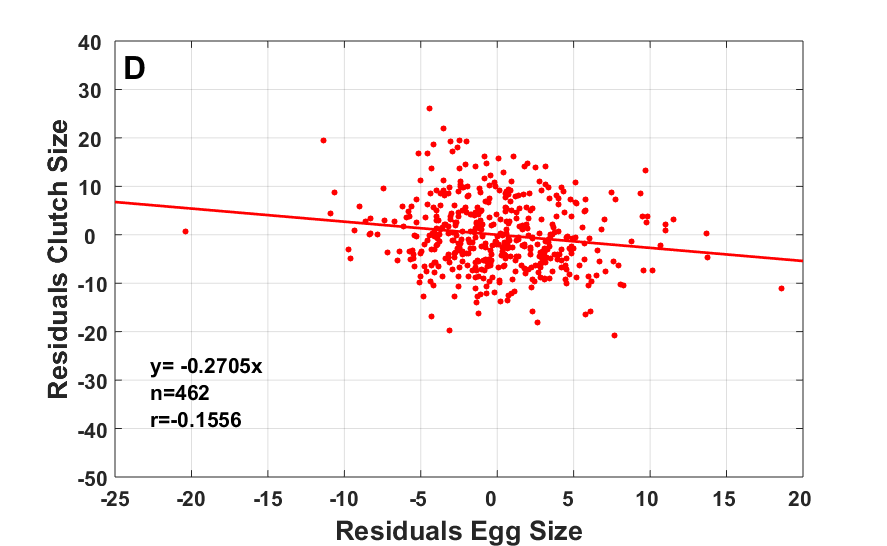 |
| --- | --- |
| 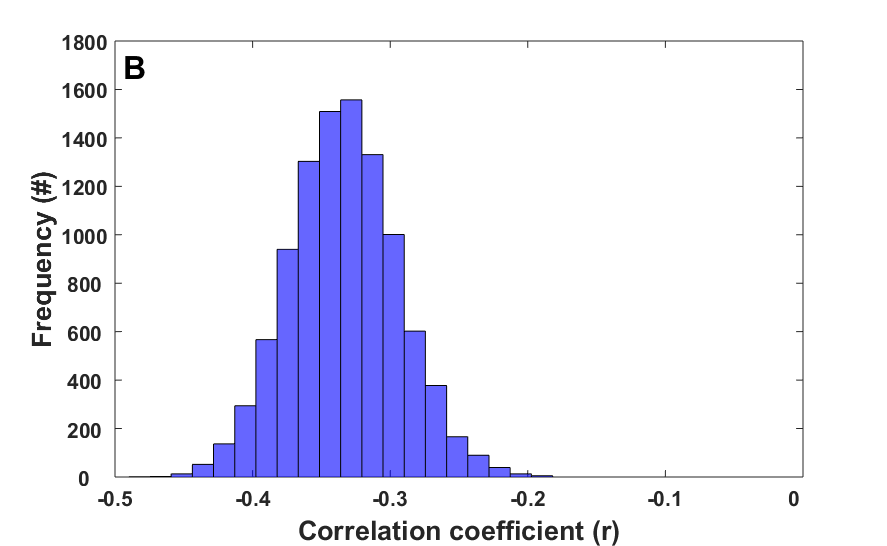 | 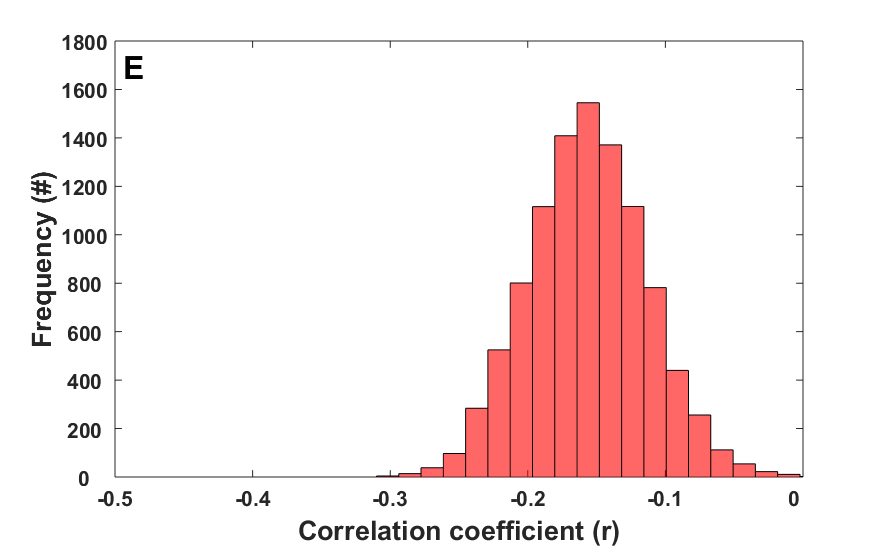 |
| 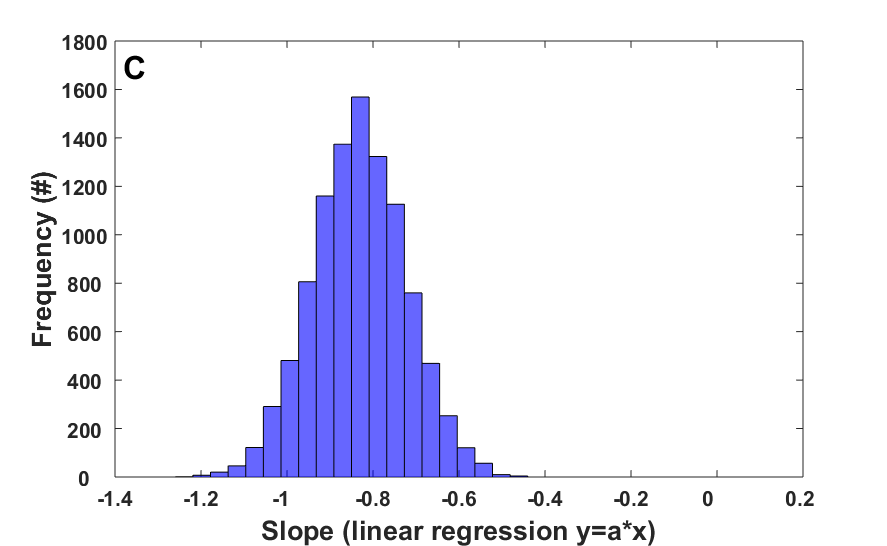 | 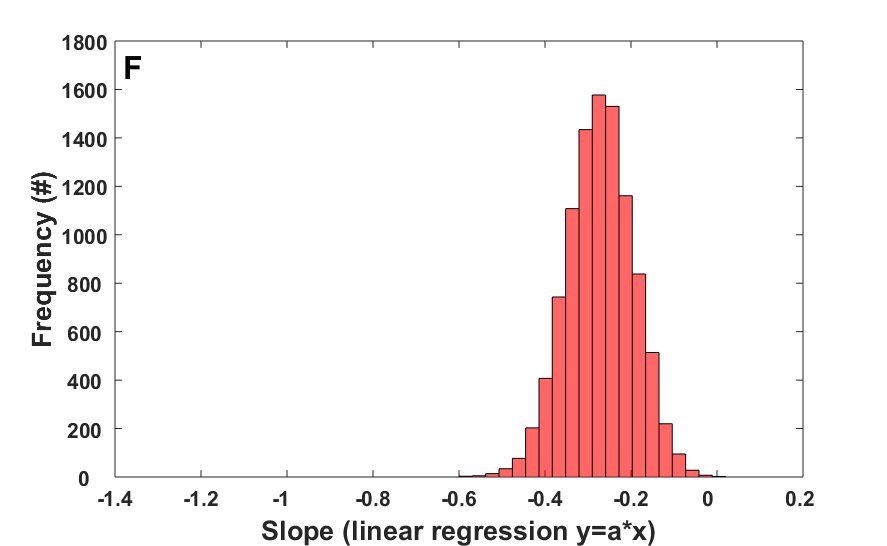 |

**SFigure1.** Relationships between residuals of linear regressions of mean egg diameter and clutch size against female prosome length applied to all data of individual ovigerous females. First column (A, B & C) with blue colour corresponds to the initial conditions and the second column (D, E & F) with red colour corresponds to the final conditions. Histograms of the distributions of the 10000 values of coefficient of correlation (*r*) generated by the resampling bootstrap method applied to initial (B) and final (E) conditions. Histograms of the distribution of the 10000 values of the slope parameter of the linear regression y=a*x for initial (C) and final (F) conditions
